# Association between the Hypertriglyceridemic Waist Phenotype, Prediabetes, and Diabetes Mellitus in Rural Chinese Population: A Cross-Sectional Study

**DOI:** 10.3390/ijerph13040368

**Published:** 2016-03-25

**Authors:** Shuang Chen, Xiaofan Guo, Shasha Yu, Guozhe Sun, Zhao Li, Yingxian Sun

**Affiliations:** Department of Cardiology, the First Affiliated Hospital of China Medical University, Shenyang 110000, China; loscs@126.com (S.C.); guoxiaofan1986@foxmail.com (X.G.); yidasasa@foxmail.com (S.Y.); gzhsun66@163.com (G.S.); meilichian@aliyun.com (Z.L.)

**Keywords:** hypertriglyceridemic waist phenotype, prediabetes, diabetes, the rural area of China

## Abstract

*Background*: The objective was to evaluate the association of the hypertriglyceridemic waist (HTGW) phenotype with prediabetes and diabetes (DM) in rural Chinese population. *Methods*: In a cross-sectional study, 11,579 adults (5361 men and 6218 women) aged 35 years or older were recruited from rural areas of China. Anthropometric measurements, laboratory examinations and self-reported information were collected by trained personnel. The HTGW phenotype was defined as elevated triglycerides and elevated waist circumference. We used logistic regression analysis to evaluate the associations of interest. *Results*: Adults with the HTGW phenotype had a significantly higher prevalence of prediabetes and diabetes than those without the HTGW phenotype. Compared with the normal waist-normal triglycerides (NWNT) group, those in the HTGW group had a higher adjusted odds ratio of diabetes (OR: 2.10; 95% CI: 1.62–2.73). The association for diabetes was stronger for men (OR: 2.27; 95% CI: 1.52–3.40) than for women (OR: 1.86; 95% CI: 1.32–2.63). However, multivariate analysis indicated that the HTGW phenotype was not associated with prediabetes. *Conclusions*: This study demonstrated that the HTGW phenotype was associated with diabetes in a large rural Chinese population, and suggested this phenotype as a simple screening tool to identify adults with cardiometabolic conditions.

## 1. Introduction

Cardiovascular disease (CVD) is one of the major causes of death in China [1]. Prediabetes and diabetes are the major risk factors for CVD. Recently, the International Diabetes Federation (IDF) estimated that the number of individuals with diabetes worldwide will be 552 million by 2030 [2]. Diabetes is a worldwide public health problem and needs urgent attention and management. In China, the enormous population, rapid economic development, lifestyle changes and aging of the population have been regarded as contributing factors to the rise of diabetes. The latest data show that the prevalence of diabetes and prediabetes in the rural Chinese population has been elevated to 10.6% and 13.0%, respectively [3].

As diabetes is becoming a serious public health issue, using a simple and inexpensive screening method for early diagnosis is particularly important [4,5]. The Third Report of the National Cholesterol Education Program Expert Panel (NCEP ATP III) indicated that abdominal obesity is independently associated with diabetes and measuring waist circumference (WC) is an effective tool to screen individuals at high risk of diabetes [6,7]. However, increased WC alone is not sufficient to discriminate intra-abdominal from subcutaneous abdominal adiposity. The elevated triglyceride level has been adopted as a marker of visceral obesity [8,9,10]. Therefore, Lemieux *et al.* [10] showed that the hypertriglyceridemia and elevated WC, known as the hypertriglyceridemic waist (HTGW) phenotype, could also predict cardiometabolic abnormalities in individuals with excess visceral adipose. The HTGW phenotype represents a simple and inexpensive surrogate marker of visceral adiposity. In previous studies, the HTGW phenotype had been indicated as a stronger predictor of CVD and diabetes than metabolic syndrome [8,11].

Although hypertriglyceridemia and elevated WC have been known as risk factors for diabetes, few studies have been performed on the relationship between the HTGW phenotype, prediabetes and diabetes. Moreover, limited data on the diabetes epidemic is available in the rural population of China. Therefore, we conducted this cross-sectional study to evaluate whether the HTGW phenotype, as the phenotype of visceral obesity, was closely associated with the risk of prediabetes and diabetes among rural Chinese adults.

## 2. Materials and Methods

### 2.1. Study Population

From July 2012 to August 2013, a representative general population aged ≥35 years was selected to describe the prevalence, incidence and natural history of cardiovascular risk factors in rural areas of Liaoning Province, and it is called the Northeast China Rural Cardiovascular Health Study (NCRCHS). The study adopted a multi-stage, stratified, random cluster-sampling scheme. In the first stage, three counties (Dawa, Zhangwu, and Liaoyang County) were randomly selected from Liaoning Province. In the second stage, one town was randomly selected from each county (a total of three towns). In the third stage, eight to 10 rural villages from each town were randomly selected (a total of 26 rural villages). All the eligible permanent residents aged ≥35 years from each village were invited to attend the study (a total of 14,016 participants). Of those, 11,956 participants agreed and completed the present study and the response rate was 85.3%. The study was approved by the Ethics Committee of China Medical University (Shenyang, China) (AF-SDP-07-1, 0-01). All procedures were performed in accordance with the ethical standards. Written consent was obtained from all participants after they had been informed of the objectives, benefits, medical items and confidentiality agreement for personal information. If the participants were illiterate, we obtained the written informed consents from their proxies. This study included subjects who had previously been diagnosed with diabetes and were under treatment for diabetes for multiple risk factors adjustment. In this report, we used data of baseline and only analyzed participants with a complete set of data in this study; we obtained a final sample size of 11,579 (5361 men and 6218 women).

### 2.2. Data Collection

Data was collected during a single clinic visit by cardiologists and trained nurses using a standard questionnaire during a face-to-face interview. Before the survey was performed, we invited all eligible investigators to attend the organized training. The training contents included the purpose of this study, how to administer the questionnaire, the standard method of measurement, the importance of standardization, and the study procedures. A strict test was evaluated after this training, and only those who scored perfectly on the test were included in the study. During data collection, our inspectors had further instructions and support.

Data on demographic characteristics, lifestyle risk factors, family income, and medical history were obtained by interview with a standardized questionnaire. There was a central steering committee with a subcommittee for quality control. Educational level was divided into primary school or below, middle school, and high school or above. Family income was classified as ≤5000, 5000–20,000 and >20,000 Chinese yuan/year. The smoking and alcohol consumption status were also surveyed. Current smokers were defined as people who were currently smoking and current drinkers were defined as people who were currently drinking. Physical activity included occupational and leisure-time physical activity. Occupational and leisure-time physical activity were merged and regrouped into the following three categories: (1) low—subjects who reported light levels of both occupational and leisure-time physical activity; (2) moderate—subjects who reported moderate or high levels of either occupational or leisure-time physical activity; and (3) high—subjects who reported a moderate or high level of both occupational and leisure-time physical activity.

### 2.3. Anthropometrics and Blood Pressure Measurements

According to the American Heart Association protocol, blood pressure was measured three times at 2 min intervals after at least 5 min of rest using a standardized automatic electronic sphygmomanometer (HEM-741C; Omron, Tokyo, Japan). The calibration of the Omron device was checked using a standard mercury sphygmomanometer every month by two doctors according to the British Hypertension Society protocol [12]. The participants were advised to avoid caffeinated beverages and exercise for at least 30 min before the measurement. During the measurement, the participants were seated with the arm supported at the level of the heart. The mean of three blood pressure measures was calculated and used in all analyses.

Weight and height were measured to the nearest 0.5 kg and 0.1 cm, respectively, with the participants in light-weight clothing and without shoes. Waist circumstance (WC) was measured at the midpoint between the lower rib and upper margin of the iliac crest using a non-elastic tape (to the nearest 0.1 cm), with the participants standing at the end of normal expiration. The body mass index (BMI) was calculated as weight in kilograms divided by the square of the height in meters.

### 2.4. Biochemical Measurements

Fasting blood samples were collected in the morning after at least 12 h of fasting for all participants. Blood samples were obtained from an antecubital vein into Vacutainer tubes containing EDTA. Serum was subsequently isolated from the whole blood, and all serum samples were frozen at −20 °C for testing at a central, certified laboratory. Fasting plasma glucose (FPG), total cholesterol (TC), low-density lipoprotein cholesterol (LDL-C), high-density lipoprotein cholesterol (HDL-C), triglyceride (TG) and other routine blood biochemical indexes were analyzed enzymatically on an Olympus AU640 auto analyzer (Olympus, Kobe, Japan). All laboratory equipment was calibrated and blinded duplicate samples were used.

### 2.5. Definitions

According to the JNC-7 report [13], hypertension was defined as systolic blood pressure (SBP) ≥ 140 mm Hg or diastolic blood pressure (DBP) ≥ 90 mm Hg or the use of antihypertensive medications. BMI was categorized into three groups: normal (BMI < 25 kg/m^2^), overweight (25 ≤ BMI < 30 kg/m^2^), and obese (BMI ≥ 30 kg/m^2^), according to the WHO criteria [14]. Diabetes was diagnosed according to the WHO criteria [15]: fasting plasma glucose ≥ 7 mmol/L (126 mg/dL) or being on treatment for diabetes. According to the American Diabetes Association (ADA) 2010 diagnostic criteria [16], impaired fasting glucose (IFG) (5.6 ≤ fasting glucose < 7.0 mmol/L) is an indicator of prediabetes. Participants were categorized into three phenotype groups based on the following cut-off points: (1) NWNT (normal waist-normal triglyceride: waist circumference <90 cm for men and <80 cm for women; serum triglyceride concentration <1.7 mmol/L); (2) EWNT/NWET (enlarged waist-normal triglycerides: waist circumference ≥90 cm for men and ≥80 cm for women; serum triglyceride concentration <1.7 mmol/L/normal waist-elevated triglycerides: waist circumference <90 cm for men and <80 cm for women; serum triglyceride concentration ≥1.7 mmol/L); (3) HTGW (hypertriglyceridemic-waist, waist circumference ≥90 cm for men and ≥80 cm for women; serum triglyceride concentration ≥1.7 mmol/L).

### 2.6. Statistical Analysis

The population was characterized through descriptive analysis, using a proportion for categorical data and mean (standard deviation, SD) for continuous variables. Continuous variables were compared among the phenotype groups by using the Analysis of Variance (ANOVA) test. The χ^2^-test analyses were used to examine associations between the categorical variables and phenotype groups. The associations between the HTGW phenotype, prediabetes, and diabetes were tested using multivariable logistic regression models, with odds ratios (ORs) and 95% confidence intervals (CIs) calculated. All statistical analyses were performed using SPSS version 19.0 software (SPSS Inc, Chicago, IL, USA), and *p* < 0.05 indicated statistical significance.

## 3. Results

Of the 11,579 adults, 6878 (59.4%) were the NWNT phenotype, 3382 (29.2%) were EWNT/NWET, and 1319 (11.4%) were HTGW. Table 1 compares the baseline characteristics of participants in each phenotype group. Participants with the HTGW phenotype were older and more likely to be females. They also reported a lower frequency of current smoking status and current drinking status, but a higher frequency of high-intensity physical activity compared with subjects in the NWNT group (*p* < 0.05).

Table 2 presents the clinical characteristics and laboratory data stratified by the three phenotype groups. Compared with subjects in the NWNT phenotype group, participants in the groups of EWNT/NWET and HTGW phenotypes tended to have higher BMI, WC, SBP, DBP, FPG, TC, TG, LDL-C and lower HDL-C (*p* < 0.001). Subjects with the HTGW phenotype also had higher prevalence of overweight, obesity, hypertension, DM, history of cardiovascular diseases and family history of diabetes than the NWNT phenotype.

The logistic regression models showed that after adjusting for age, subjects with the HTGW phenotype had (OR: 4.94; 95% CI: 4.20–5.82) in the total population, (OR: 4.83; 95% CI: 3.76–6.20) in men and (OR: 4.72; 95% CI: 3.79–5.88) in women of DM, respectively, compared to subjects with the NWNT phenotype (Table 3). After adjusting for age, sex, smoking, drinking, education level, physical activity, BMI, hypertension, history of cardiovascular disease, and family history of DM, the relationship between DM and the HTGW phenotype remained significant, with (OR: 2.10; 95% CI: 1.62–2.73) for the total population, (OR: 2.27; 95% CI: 1.52–3.40) for men and (OR: 1.86; 95% CI: 1.32–2.63) for women. Among subjects with EWNT/NWET, the strength of the association for DM was attenuated but remained statistically significant (OR: 1.59; 95% CI: 1.31–1.94).

Then, Table 4 shows the risk of prediabetes according to the three phenotype groups by multivariate logistic regression analysis. In the age-adjusted model, subjects with the HTGW phenotype were more likely (OR: 1.24; 95% CI: 1.10–1.40) to have prediabetes than subjects from the NWNT group, and also for men (OR: 1.26; 95% CI: 1.05–1.51) and women (OR: 1.22; 95% CI: 1.03–1.43), respectively. However, in the multivariable model, there existed no association between the HTGW phenotype and prediabetes.

## 4. Discussion

The major finding of this current study was the higher prevalence odds of DM associated with the HTGW phenotype in this rural Chinese population. It is the first study assessing the association of the HTGW phenotype with prediabetes and DM in the rural area of China. The relationship between the HTGW phenotype and DM was strong and still remained significant after adjusting for the effect of covariates, such as demographic characteristics, lifestyles, and family history of DM. This result was consistent with but had a lower strength of association than a Canadian study which also found that the HTGW phenotype was significantly associated with DM (OR: 4.96; 95% CI: 2.49–9.88) [17]. In our study, multivariable analysis showed that the strength of the relationship between the HTGW phenotype and DM was quite similar in both sexes (OR: 2.27; 95% CI: 1.52–3.40 for men and OR: 1.86; 95% CI: 1.32–2.63 for women). Okosun *et al.* [18] also observed a significant association of the HTGW phenotype and DM in American adults, in which the Black women with the HTGW phenotype had quite higher odds (OR: 5.62; 95% CI: 1.04–9.42) of DM compared to their male counterparts (OR: 3.94; 95% CI: 2.85–3.90). Our study indicated that no association existed between the HTGW phenotype and prediabetes. Until now, only one study in Chinese urban adults had been conducted to assess the association of the HTGW phenotype and prediabetes [18]. This study of the Chinese urban population indicated that only women with the HTGW phenotype were at higher risk for prediabetes (OR: 1.51; 95% CI: 1.04–2.19) compared with subjects in the NWNT phenotype group.

Data from this study indicated a positive and significant association between the HTGW phenotype and DM in the rural Chinese population, but no association with prediabetes. Although the EWNT/NWET phenotype had a 1.59-fold increase in DM risk after adjustment for covariates, the OR was lower than in the HTGW group (2.10). Our results provided evidence that both HTGW and EWNT/NWET phenotypes are simple and inexpensive markers to identify subjects who are at increased risk of diabetes, and the HTGW phenotype is the better and stronger one. To our best knowledge, the mechanism behind the association observed with the HTGW phenotype and DM remains unclear. The central pattern of body fat distribution, particularly an increased amount of visceral adipose tissue, has been independently associated with DM [19]. Visceral adipose tissue (VAT) increases the release of free fatty acids into the portal vein which can induce insulin resistance and inhibit the uptake of glucose [20]. Although an increased secretion of insulin compensates temporarily for the alterations, the continuously increased level of glucose may promote the incidence of DM [21]. In 2003, a study from Italy indicated that a genetic predisposition to type 2 diabetes, probably in association with slightly elevated glucose levels, may accelerate the development of atherosclerosis and increase the risk for coronary heart disease in glucose-tolerant individuals [21]. Ciccone MM and Scicchitano Precently pointed out the tight relationship between nutraceuticals and cardiovascular disease, such as dyslipidemia and diabetes [21].

It is well known that the gold standard for quantitative assessment of visceral adiposity is imaging techniques such as magnetic resonance imaging (MRI) and computed tomography (CT). However, the exposure to radiation and the high costs limit the use of imaging methods for research and clinical practice. An elevated WC alone does not identify individuals with an excess amount of VAT, because the accumulation of adipose tissue can be subcutaneous [8]. Alternatively, the HTGW phenotype was suggested to offer a simple and inexpensive method to discriminate between visceral adiposity and subcutaneous adiposity [22]. Thus, the HTGW phenotype, as a practical and easily applied tool, is a useful approach to identify subjects with VAT compared with the phenotypes of EWNT and NWET [23,24]. For the HTGW phenotype, as a phenotype associated with DM in adults, the low cost makes it an available and easily applicable indicator in clinical practice and research. In the present study, the HTGW phenotype remained significantly associated with DM in the rural Chinese population even after adjustment for covariates. Moreover, our study showed higher levels of fasting glucose in the rural Chinese population with the HTGW phenotype compared with their counterparts with EWNT/NWET or NWNT. Thus, more attention should be paid to adults with HTGW among this population. Our results also indicated that the HTGW phenotype was not a risk factor for prediabetes.

Study strengths and limitations: Several limitations in this study need to be acknowledged. First, because of its cross-sectional design, we were unable to determine whether or not there was a causal association between the HTGW phenotype, prediabetes, and diabetes mellitus. Thus, the obtained associations in this study should be considered with caution. Second, despite extensive adjustment that has been done in our study, the possibility still exists that unmeasured confounders may explain part of the association between the HTGW phenotype and diabetes mellitus. However, the strengths of this study are its population-based design, large sample size, and the extensive information on confounders. Our study provided strong evidence of a relationship between the HTGW phenotype and diabetes mellitus. These data are particularly important in the case of adults in the rural Chinese population who have a high absolute risk of cardiovascular diseases. It will be necessary to confirm the effect of the HTGW phenotype on predicting diabetes mellitus in a future longitudinal study, and replication of the results in further investigations with a large-scale population is necessary before firm conclusions can be drawn.

## 5. Conclusions

In summary, our present study reported that the HTGW phenotype may be a useful marker for identifying individuals at risk of DM. Because measurements of WC and fasting serum TG concentrations are relatively inexpensive and simple to conduct in a clinical setting, using the HTGW to identify individuals at high risk of CVD and DM who may benefit from early intervention has important public health implications for better prevention, diagnosis, and treatment. Due to the growing prevalence of obesity and DM worldwide, more attention should be paid to developing simple and inexpensive indicators for the early identification of individuals at substantial risk of progressing to cardiometabolic abnormalities. However, further prospective investigations are necessary to understand the predictive usefulness of the HTGW phenotype as a DM risk indicator.

## Figures and Tables

**Table 1:** Baseline characteristics of the participants in each phenotype group (*n* = 11,579).

Variables	NWNT	EWNT/NWET	HTGW	*p* Value
	(*n* = 6878)	(*n* = 3382)	(*n* = 1319)	
**Age, year**	53.4 ± 10.7	54.3 ± 10.4 *	54.8 ± 10.0 †	<0.001
**Sex,%**				<0.001
Male	3376 (49.1)	1433 (42.4) *	552 (41.8) †	
Female	3502 (50.9)	1949 (57.6) *	767 (58.2) †	
**Race,%**				0.641
Han	6528 (94.9)	3202 (94.7)	1244 (94.3)	
Others	350 (5.1)	180 (5.3)	75 (5.7)	
**Current smoking status, %**	2580 (37.5)	1078 (31.9) *	431 (32.7) †	<0.001
**Current drinking status, %**	1606 (23.3)	743 (22.0)	269 (20.4) †	0.036
**physical activity, %**				<0.001
low	1894 (27.5)	1066 (31.5) *	483 (36.6) †	
moderate	4619 (67.2)	2124 (62.8) *	740 (56.1) †	
high	365 (5.3)	192 (5.7) *	96 (7.3) †	
**Education, %**				
Primary school or below	3309 (48.1)	1747 (51.7) *	711 (53.9) †	<0.001
Middle school	2929 (42.6)	1321 (39.1) *	469 (35.6) †	
High school or above	640 (9.3)	314 (9.3) *	139 (10.5) †	
**Family income (CNY/year), %**				0.482
<5000	842 (12.2)	448 (13.2)	152 (11.5)	
5000–20,000	3759 (54.7)	1819 (53.8)	732 (55.5)	
>20,000	2277 (33.1)	1115 (33.0)	435 (33.0)	

Data are expressed as the mean ± SD or as n (%). Abbreviations: NWNT, normal waist-normal triglycerides; EWNT/NWET, enlarged waist-normal triglycerides/normal waist-elevated triglycerides; HTGW, hypertriglyceridemic waist; CNY, China Yuan (1CNY = 0.161 USD). * *p* < 0.05 between NWNT and EWNT/NWET; † *p* < 0.05 between HTGW and NWNT.

**Table 2:** Baseline clinical characteristics and laboratory data of the participants in each phenotype group (*n* = 11,579).

Variables	NWNT	EWNT/NWET	HTGW	*p* Value
	(*n* = 6878)	(*n* = 3382)	(*n* = 1319)	
BMI (kg/m^2^)	23.1 ± 2.8	26.7 ± 3.4 *	28.6 ± 3.1 #†	<0.001
Overweight, %	1643 (23.9)	2376 (70.3) *	1191 (90.3) #†	<0.001
Obesity, %	31 (0.5)	504 (14.9) *	364 (27.6) #†	<0.001
WC, cm	77.1 ± 6.8	88.6 ± 8.4 *	94.3 ± 6.5 #†	<0.001
SBP, mm Hg	137.9 ± 22.3	146.4 ± 24.0 *	150.4 ± 23.3 #†	<0.001
DBP, mm Hg	80.0 ± 11.2	84.3 ± 11.7 *	87.0 ± 12.2 #†	<0.001
Hypertension,%	2932 (42.6)	2049 (60.6) *	938 (71.1) #†	<0.001
TC, mmol/L	5.0 ± 1.0	5.4 ± 1.1 *	5.8 ± 1.3 #†	<0.001
TG, mmol/L	1.1 ± 0.4	2.0 ± 1.7 *	3.5 ± 2.4 #†	<0.001
HDL-C, mmol/L	1.5 ± 0.4	1.3 ± 0.3 *	1.2 ± 0.3 #†	<0.001
LDL-C, mmol/L	2.8 ± 0.7	3.1 ± 0.9 *	3.3 ± 0.9 #†	<0.001
FPG, mmol/L	5.7 ± 1.2	6.1 ± 1.8 *	6.6 ± 2.3 #†	<0.001
Blood glucose level (%)				<0.001
Normal (<5.6 mmol/L)	4086 (59.4)	1557 (46.0) *	481 (36.5) #†	
Pre-diabetes (5.6–7.0 mmol/L)	2426 (35.3)	1385 (41.0) *	538 (40.8) #†	
Diabetes (>7.0 mmol/L)	366 (5.3)	440 (13.0) *	300 (22.7) #†	
DM, %	408 (5.9)	475 (14.0) *	317 (24.0) #†	<0.001
History of CVD, %	853 (12.4)	579 (17.1) *	280 (21.2) #†	<0.001
Family history of diabetes, %	517 (7.5)	355 (10.5) *	182 (13.8) #†	<0.001

Data are expressed as the mean ± SD or as n (%). Abbreviations: NWNT, normal waist-normal triglycerides; EWNT/NWET, enlarged waist-normal triglycerides/normal waist-elevated triglycerides; HTGW, hypertriglyceridemic waist; BMI, body mass index; WC, waist circumference; SBP, systolic blood pressure; DBP, diastolic blood pressure; TC, total cholesterol; TG, triglyceride; LDL-C, low-density lipoprotein cholesterol; HDL-C, high-density lipoprotein cholesterol; FPG, fasting plasma glucose; DM, diabetes mellitus; CVD, cardiovascular disease. * *p* < 0.05 between NWNT and EWNT/NWET, # *p* < 0.05 between EWNT/NWET and HTGW, † *p* < 0.05 between HTGW and NWNT.

**Table 3:** Risk of diabetes according to the three phenotype groups for men and women.

Phenotype	Age-Adjusted Model			Multivariable Model		
	OR	95% CI	*p*	OR	95% CI	*p*
Total						
NWNT	1.00			1.00		
EWNT/NWET	2.56	2.22–2.94	<0.001 *	1.59	1.31–1.94	<0.001 *
HTGW	4.94	4.20–5.82	<0.001 *	2.10	1.62–2.73	<0.001 *
Men						
NWNT	1.00			1.00		
EWNT/NWET	2.61	2.13–3.21	<0.001 *	1.62	1.20–2.17	0.002 *
HTGW	4.83	3.76–6.20	<0.001 *	2.27	1.52–3.40	<0.001 *
Women						
NWNT	1.00			1.00		
EWNT/NWET	2.42	2.00–2.94	<0.001 *	1.51	1.17–1.97	0.002 *
HTGW	4.72	3.79–5.88	<0.001 *	1.86	1.32–2.63	<0.001 *

Abbreviations: NWNT, normal waist-normal triglycerides; EWNT/NWET, enlarged waist-normal triglycerides/normal waist-elevated triglycerides; HTGW, hypertriglyceridemic waist. Multivariable model: adjusted for age, sex, smoking, drinking, education level, physical activity, BMI, hypertension, history of cardiovascular disease, and family history of DM. * *p* < 0.05.

**Table 4:** Risk of prediabetes according to the three phenotype groups for men and women.

Phenotype	Age-Adjusted Model			Multivariable Model		
	OR	95% CI	*p*	OR	95% CI	*p*
Total						
NWNT	1.00			1.00		
EWNT/NWET	1.26	1.15–1.37	<0.001 *	1.15	1.01–1.31	0.032 *
HTGW	1.24	1.10–1.40	0.001 *	1.03	0.85–1.25	0.789
Men						
NWNT	1.00			1.00		
EWNT/NWET	1.21	1.07–1.38	0.002 *	1.13	0.93–1.37	0.214
HTGW	1.26	1.05–1.51	0.013 *	1.05	0.78–1.40	0.753
Women						
NWNT	1.00			1.00		
EWNT/NWET	1.31	1.17–1.48	<0.001 *	1.136	0.95–1.36	0.163
HTGW	1.22	1.03–1.43	0.021 *	0.952	0.73–1.24	0.713

Abbreviations: NWNT, normal waist-normal triglycerides; EWNT/NWET, enlarged waist-normal triglycerides/nor mal waist-elevated triglycerides; HTGW, hypertriglyceridemic waist. Multivariable model: adjusted for age, sex, smoking, drinking, education level, physical activity, BMI, hypertension, history of cardiovascular disease, and family history of DM. * *p* < 0.05.

## Abbreviations

The following abbreviations are used in this manuscript:

CVD: cardiovascular disease
OR: odds ratio
CI: confidence interval
SBP: systolic blood pressure
DBP: diastolic blood pressure
WC: waist circumference
BMI: body mass index
TC: total cholesterol
TG: triacylglycerol
HDL-C: high-density lipoprotein cholesterol
LDL-C: low-density lipoprotein cholesterol
FPG: fasting plasma glucose
DM: diabetes mellitus
NWNT: normal waist-normal triglycerides
EWNT/NWET: enlarged waist-normal triglycerides/normal waist-elevated triglycerides
HTGW: hypertriglyceridemic waist
